# Asymptomatic Glomus Tumor of the Mediastinum

**DOI:** 10.1155/2015/631625

**Published:** 2015-09-09

**Authors:** Meletios Kanakis, Nikoletta Rapti, Maria Chorti, Achilleas Lioulias

**Affiliations:** ^1^Department of Thoracic Surgery, Sismanoglio General Hospital of Athens, 1 Sismanogliou Street, Maroussi, 15126 Athens, Greece; ^2^Department of Pathology, Sismanoglio General Hospital of Athens, 1 Sismanogliou Street, Maroussi, 15126 Athens, Greece

## Abstract

Glomus tumors are rare benign neoplasms that predominate in limbs. Infrequently, they can occur in a wide anatomic distribution, to include sites not known to contain glomus cells. Although glomus tumors are usually small, pain and tenderness are common clinical symptoms. We report the case of a 69-year-old man with an asymptomatic large mediastinal glomus tumor, who underwent surgical resection.

## 1. Introduction

Glomus tumors are thought to arise from the glomus body, a specialized thermoregulatory shunt highly concentrated in the fingers and toes. Most lesions are solitary and localized to cutaneous sites; however, they may have extracutaneous involvement [1]. While the vast majority of glomus tumors are benign, malignant cases have been reported but rarely.

## 2. Case Presentation

**Figure 1:**
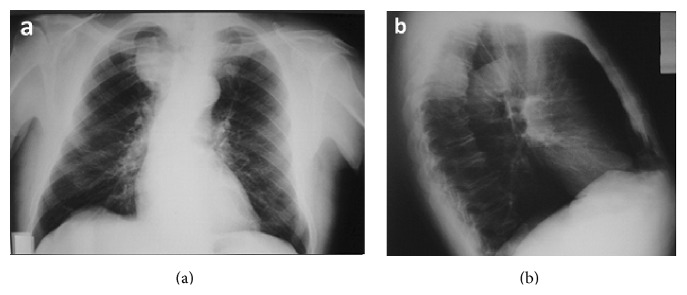
Chest X-ray film showing the tumor located to the posterior mediastinum.

**Figure 2:**
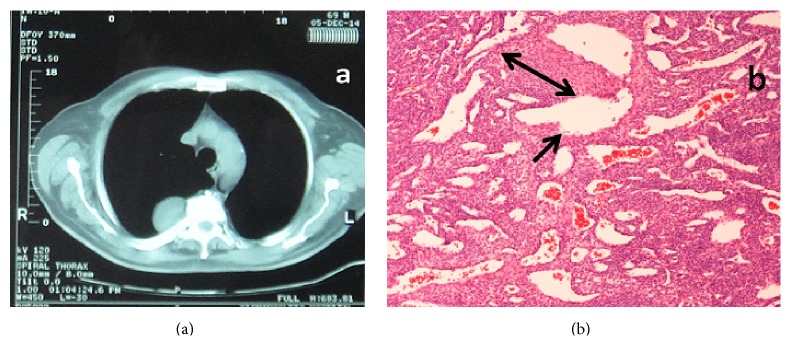
(a) Computed tomography showing the tumor to the right posterior mediastinum at the level of T3 vertebral body. (b) Solid sheets of glomus cells (double arrow) around dilated vessels (simple arrow). Hematoxylin and eosin stain, original magnification ×100.

A 69-year-old man suffered from benign prostatic hyperplasia and was hospitalized in the urology department of our hospital in order to undergo a transurethral resection of the prostate. The routine preoperative chest X-ray (Figure 1) revealed a mass in the right upper mediastinum. He underwent uneventful prostatectomy and he was referred to our department for further evaluation and management. The patient was asymptomatic and his past medical history included an orthopaedic surgery and bilateral inguinal hernia repair before seven and two years, respectively. His physical examination, his vital signs, and the laboratory studies were normal. Computed tomography (CT) identified a lesion of the right upper posterior mediastinum at the level of T3 (Figure 2(a)). CT guided biopsy was inconclusive. Differential diagnosis included mainly neurogenic origin tumors.

Our patient underwent a right posterolateral thoracotomy through the 5th intercostal space. A well-circumscribed vascular lesion was resected en bloc with macroscopically clear margins. The right thoracic cavity was drained by a single chest tube 32F. The patient made an uneventful postoperative recovery and he was discharged on the fifth postoperative day. Pathology report showed a benign glomus tumor of the mediastinum measuring 4.5 × 4 × 2.5 cm, with clear resection margins (Figure 2(b)). The patient, 16 months after surgery, remained in an excellent clinical status with no evidence of recurrence.

## 3. Discussion

Glomus body is specialized arteriovenous anastomosis found most often in the fingers and is characterized by an afferent arteriole, Sucquet-Hoyer canal, and an efferent venule. Glomus cells surround this arteriovenous anastomosis, due to their ability of contraction, which play an important role in thermoregulation [1, 2]. Interestingly, while glomus tumors predominate on the digits or the deep dermis of the palm and sole, these tumors can occur in a wide anatomic distribution, to include sites not known to contain glomus cells. One explanation for this finding is that these tumors may arise from perivascular smooth muscle cells that can differentiate into glomoid cells [2]. Extracutaneous sites that have been reported are involvement of the gastrointestinal tract, trachea, nerve, bone, mediastinum, liver, pancreas, kidney, ovary, lung, and penis [1, 2].

Mediastinal location is very rare. To the best of our knowledge, six cases have been described in the English literature so far [3–8].

Glomus tumors are typically composed of 3 components: glomus cells, vasculature, and smooth muscle cells. They may be subcategorized as solid glomus tumor (with poor vasculature and scant smooth muscle component), glomangioma (with prominent vascular component), or glomangiomyoma (with prominent vascular and smooth muscle components). Solid glomus tumor is the most common variant (75%) followed by glomangioma (20%) and glomangiomyoma (5%) [2, 3, 9].

Glomus tumors consist of branching vascular channels lined by endothelial cells, interspersed by round glomus cells forming nests, sheets, and trabeculae [2]. Differential diagnosis of glomus tumor includes mainly carcinoid tumor, paraganglioma, solitary fibrous tumor, and leiomyoma. Carcinoid tumors, although they have similar morphology, are positive for cytokeratin, chromogranin, and synaptophysin. Paragangliomas are mainly composed of round epithelioid cells and express neuroendocrine markers and S-100. Solitary fibrous tumors consist of spindle cells and they are positive for vimentin and CD34 in contrast with glomus tumors. Leiomyomas and other smooth muscle neoplasms consist of spindle cells with a fascicular pattern and express smooth muscle markers such as actin and h-caldesmon [2–9].

Most glomus tumors are solitary and sporadic, but some cases of glomus tumor are multiple. These disseminated variants of glomus tumor, known as glomangiomas, differ clinically from solitary glomus tumors and have been linked to chromosome 1p21-22 and are caused by truncating mutations in glomulin [10]. The pattern of inheritance is autosomal dominant. Note that most cases of glomangiomas manifest as sporadic tumors; however, familial cases with autosomal dominant inheritance patterns have also been described.

In rare cases, malignant transformation within glomus tumors, which produce surrounding tissues infiltration, has been described [3]. Metastases have also been described [11]. In 2001, Folpe et al. [12] suggested the following histopathologic criteria for malignancy in glomus: tumor large size (2.0 cm) and deep location or moderate to high nuclear grade and increased mitotic rate (5 per 50 high-power fields) or the presence of atypical nuclei. If these histologic criteria of malignancy are met, the risk of metastases exceeds 25% [2, 12].

The most reported symptoms of glomus tumors were pain, local tenderness, and cold hypersensitivity mainly in cutaneous lesions. Rarely, patients are asymptomatic and glomus tumor is an incidental finding [1, 8]. Usually glomus tumors are small, rarely exceeding 1 cm [2]. Our patient was asymptomatic and the lesion was almost 5 cm. Diagnosis is a challenge, especially in the cases of extradigital lesions. In our case a plain chest X-ray revealed a mass in the upper mediastinum and led to a CT scan. Magnetic resonance imaging, however, is an excellent examination tool for detecting the soft-tissue origin of a glomus tumor as small as 2 mm. This method can also accurately define the location and limits of a tumor [13].

The treatment of choice for solitary glomus tumors is total surgical excision with clear macroscopic margins, which is curative. Benign lesions do not need adjuvant therapy.

The prognosis for patients with glomus tumors is excellent. Excision of painful lesions most often results in cure, with a low recurrence rate for solitary lesions.

## References

[1] Schiefer T. K., Parker W. L., Anakwenze O. A., Amadio P. C., Inwards C. Y., Spinner R. J. (2006). Extradigital glomus tumors: a 20-year experience. *Mayo Clinic Proceedings*.

[2] Gombos Z., Zhang P. J. (2008). Glomus tumor. *Archives of Pathology and Laboratory Medicine*.

[3] Choi Y. J., Yang K. H., Gang S. J., Kim B. K., Kim S. M. (1991). Malignant glomus tumor originating in the superior mediastinum—an immunohistochemical and ultrastructural study. *Journal of Korean Medical Science*.

[4] Gaertner E. M., Steinberg D. M., Huber M. (2000). Pulmonary and mediastinal glomus tumors: report of five cases including a pulmonary glomangiosarcoma: a clinicopathologic study with literature review. *The American Journal of Surgical Pathology*.

[5] Brindley G. V. (1949). Glomus tumor of the mediastinum. *The Journal of Thoracic and Cardiovascular Surgery*.

[6] Bali G. S., Hartman D. J., Haight J. B., Gibson M. K. (2013). A rare case of malignant glomus tumor of the esophagus. *Case Reports in Oncological Medicine*.

[7] Rychlik I. J., O'Donnell M. E., Davey P., Merard R., McGuigan J. (2014). Glomus tumor of the mediastinum. *Asian Cardiovascular and Thoracic Annals*.

[8] Jang S. H., Cho H. D., Lee J. H. (2015). Mediastinal glomus tumor: a case report and literature review. *Journal of Pathology and Translational Medicine*.

[9] Fletcher C. D. M., Unni K., Meretens F. (2002). *Pathology and Genetics of Tumours of the Nervous System*.

[10] Brouillard P., Ghassibé M., Penington A. (2005). Four common glomulin mutations cause two thirds of glomuvenous malformations (‘familial glomangiomas’): evidence for a founder effect. *Journal of Medical Genetics*.

[11] Watanabe K., Sugino T., Saito A., Kusakabe T., Suzuki T. (1998). Glomangiosarcoma of the hip: report of a highly aggressive tumour with widespread distant metastases. *British Journal of Dermatology*.

[12] Folpe A. L., Fanburg-Smith J. C., Miettinen M., Weiss S. W. (2001). Atypical and malignant glomus tumors: analysis of 52 cases, with a proposal for the reclassification of glomus tumors. *The American Journal of Surgical Pathology*.

[13] Ham K. W., Yun I. S., Tark K. C. (2013). Glomus tumors: symptom variations and magnetic resonance imaging for diagnosis. *Archives of Plastic Surgery*.

